# Identification of a novel type II-C Cas9 from the fish pathogen *Flavobacterium psychrophilum*

**DOI:** 10.3389/fmicb.2023.1181303

**Published:** 2023-06-15

**Authors:** Fuguang Chen, Di Wang, Tongyan Lu, Shaowu Li

**Affiliations:** ^1^Department of Aquatic Animal Health, Heilongjiang River Fisheries Research Institute, Chinese Academy of Fishery Sciences, Harbin, China; ^2^Key Laboratory of Aquatic Animal Diseases and Immune Technology of Heilongjiang Province, Harbin, China

**Keywords:** *Flavobacterium psychrophilum*, salmonids, Cas9, bacteriophage, interference, origin, evolution

## Abstract

*Flavobacterium psychrophilum* is the causative agent of rainbow trout fry syndrome and bacterial cold-water disease in salmonid fish worldwide. As an important fish pathogen, *F. psychrophilum* is frequently exposed to multiple invading genetic elements in natural environments. Endonuclease Cas9 provides bacteria with adaptive interference against invading genetic elements. Previous studies revealed that several *F. psychrophilum* strains harbored a type II-C Cas9 called Fp1Cas9, but little is known about the potential role of this endonuclease against invading genetic elements. In this work, we identified a gene encoding a novel type II-C Cas9 called Fp2Cas9 from *F. psychrophilum* strain CN46. Through bacterial RNA sequencing, we demonstrated active transcription of both Fp2Cas9 and pre-crRNAs in strain CN46. Bioinformatics analysis further revealed that the transcription of Fp2Cas9 and pre-crRNAs was driven by a newly integrated promoter sequence and a promoter element embedded within each CRISPR repeat, respectively. To formally demonstrate that Fp2Cas9 and associated crRNAs yielded functional interference in strain CN46, a plasmid interference assay was performed, resulting in adaptive immunity to target DNA sequences in *Flavobacterium* bacteriophages. Phylogenetic analysis demonstrated that Fp2Cas9 was present only in several *F. psychrophilum* isolates. Phylogenetic analysis revealed that this novel endonuclease was probably acquired through horizontal gene transfer from the CRISPR-Cas9 system in an unidentified *Flavobacterium* species. Comparative genomics analysis further showed that the Fp2Cas9 was integrated into the type II-C CRISPR-Cas locus in strain CN38 instead of the original Fp1Cas9. Taken together, our results shed light on the origin and evolution of Fp2Cas9 gene and demonstrated that this novel endonuclease provided adaptive interference against bacteriophage infections.

## Introduction

*Flavobacterium psychrophilum*, a bacterium belonging to the genus *Flavobacterium*, is a serious causative agent of rainbow trout fry syndrome and bacterial cold-water disease in salmonid fish. All salmonid fish reared in freshwater appear susceptible to this pathogen, especially rainbow trout (*Oncorhynchus mykiss*), coho salmon (*Oncorhynchus kisutch*), Atlantic salmon (*Salmo salar*), and ayu (*Plecoglossus altivelis*) (Duchaud et al., 2018). Since the first case of *F. psychrophilum* infection was reported in America in 1948, this pathogen has been identified in Norway, France, Germany, Denmark, Japan, Chile, China and other regions of the world involved in salmonid aquaculture (Duchaud et al., 2018; Knupp et al., 2019; Calvez et al., 2021; Li et al., 2021). Up to now, *F. psychrophilum* hampers seriously the productivity of salmonid farming worldwide. Despite several progress in the past decade (Hesami et al., 2011; Castillo et al., 2015; Barbier et al., 2020; Jørgensen et al., 2022), many aspects of the *F. psychrophilum* lifestyle, including molecular mechanisms of its adaptation to changing environments, remain poorly understood.

*F. psychrophilum* is frequently exposed to invading genetic elements in natural environments, such as bacteriophages (Stenholm et al., 2008; Kim et al., 2010; Castillo et al., 2012, 2014; Castillo and Middelboe, 2016). To survive in bacteriophage-rich water environments, *F. psychrophilum* is expected to possess efficient defense systems to regulate genetic exchanges which favored in such environments. The genetic diversity and evolution of *F. psychrophilum* isolates could reflect the continuous balance between the acquisition of adaptive traits for their survival and the efficient defense against invading genetic elements, such as bacteriophages and plasmids (Duchaud et al., 2018; Knupp et al., 2019). CRISPR-Cas (clustered regularly interspaced short palindromic repeats and CRISPR-associated proteins) systems provide adaptive immunity against invading genetic elements in bacteria and archaea (Makarova et al., 2020). To date, CRISPR-Cas systems are classified into two major classes (1 and 2) and six types (I–VI), depending on their sub-components and functions (Koonin et al., 2017; Makarova et al., 2018, 2020). Class 1 CRISPR-Cas members utilize multi-subunit effector complexes, whereas Class 2 members work with a single multidomain effector protein for DNA or RNA cleavage (Makarova et al., 2020). Class 2 CRISPR-Cas members include types II, V, and VI (Makarova et al., 2020). The distinguishing feature of these types is that their effector complexes consist of a single, large, multidomain protein (Makarova et al., 2020). For instance, type II CRISPR-Cas system contains an endonuclease well known as Cas9, including RuvC nuclease domain, HNH nuclease domain and the PAM-interacting domain (PID) (Anders et al., 2014; Nishimasu et al., 2015; Das et al., 2020). Type II CRISPR-Cas systems are presently subdivided into three subtypes: type II-A, type II-B, and type II-C if they have Csn2 or Cas4 (Makarova et al., 2017). Type II-C system is the simplest type II CRISPR-Cas system and the type II-C locus contains 5 crucial components: an endonuclease Cas9, two adaptation proteins Cas1 and Cas2, a CRISPR array, and a trans-activating CRISPR RNA (tracrRNA) (Zhang et al., 2013; Fedorova et al., 2020a,b; Hoikkala et al., 2021). The type II-C CRISPR-Cas immune response consists of three main stages: adaptation, expression and interference. At the adaptation stage, a distinct complex of Cas proteins comprised of Cas9, Cas1, and Cas2 recognizes a specific motif known as a protospacer-adjacent motif (PAM) depending on the PAM-interacting domain of Cas9, binds to a target DNA and cleaves out a portion of the target DNA called protospacers (Anders et al., 2016). PAM is typically 2–8 nucleotides long and located immediately downstream of the protospacer (Fedorova et al., 2020b). Cas1–Cas2 complex that acts as the spacer integrase integrates the protospacer DNA into the CRISPR array, resulting in simultaneous spacer insertion and repeat duplication at the 5ʹ end of the CRISPR array (McGinn and Marraffini, 2019; Hoikkala et al., 2021). At the expression stage, the CRISPR array is typically transcribed as many transcripts called the pre-CRISPR RNAs (pre-crRNAs) driven by promoter elements embedded within each repeat (Zhang et al., 2013; Fedorova et al., 2020a,b; Hoikkala et al., 2021). pre-crRNAs are further processed into mature CRISPR RNAs (crRNAs) mediated by host RNases, each of which contains the spacer sequence and the part of the 3′ flanking repeat (Zhang et al., 2013). At the interference stage, the crRNA, which typically remains bound to the processing tracrRNA-Cas9 complex, serves as a guide to recognize the protospacer in invading genetic elements. Cas9 recognizes the specific PAM sequence depending mainly on the C-terminal PAM-interacting domain similar to other type II Cas9s (Nishimasu et al., 2015; Anders et al., 2016; Yamada et al., 2017; Das et al., 2020; Xie et al., 2022). Target DNA strand complementary to crRNA is cleaved by the HNH nuclease domain of endonuclease Cas9 whereas non-target DNA strand non-complementary to crRNA is cleaved by the RuvC nuclease domain of endonuclease Cas9 (Yamada et al., 2017; Sun et al., 2019; Das et al., 2020). Thus, type II-C systems provide adaptive immunity against invading genetic elements.

In 2015, Castillo et al. reported that several *F. psychrophilum* strains harbored a type II-C endonuclease Cas9 called Fp1Cas9 (Castillo et al., 2015). However, Fp1Cas9 has not been well characterized to date and failed to provide adaptive interference against invading genetic elements. In this work, we identified a gene encoding a novel type II-C endonuclease Cas9 called Fp2Cas9 in *F. psychrophilum* strain CN46. We further demonstrated that both Fp2Cas9 gene and pre-crRNAs were actively transcribed and formally demonstrated the adaptive interference against foreign plasmids. We provide *in silico* evidence that this novel endonuclease present only in several *F. psychrophilum* strains were acquired through horizontal gene transfer from a *Flavobacterium* species and then integrated into the type II-C CRISPR-Cas locus instead of the original Fp1Cas9.

## Materials and methods

### Bacterial strains and growth conditions

*F. psychrophilum* strains used in this study were the wild-type strains. *F*. *psychrophilum* cultures were grown at 18°C in tryptone yeast extract salts (TYES) medium (Li et al., 2021), which contained, per liter, 4 g tryptone, 0.5 g yeast extract, 0.5 g MgSO_4_·7H_2_O, and 0.2 g CaCl_2_, with pH adjusted to 7.2. For solid media, agar was used at 12 g/liter unless indicated otherwise. For most experiments, *F*. *psychrophilum* strains were streaked from-80°C freezer tubes onto TYES agar and incubated for 96 to 120 h at 18°C, and then used to inoculate 10 mL TYES broth cultures, which were incubated for 48 to 72 h at 18°C with shaking at 120 rpm. *E. coli* strains were grown in lysogeny broth (LB) at 37°C (Barbier et al., 2020). If required, antibiotics were used at the following concentrations: ampicillin, 100 μg/mL for *E. coli*, and erythromycin, 10 μg/mL for *F. psychrophilum*.

### *Flavobacterium psychrophilum de novo* DNA sequencing

The three isolates were obtained between 2017 and 2020 from salmonids at different fish farms in China. A single colony of *F*. *psychrophilum* CN06, CN38, or CN46 was inoculated into TYES broth and incubated at 18°C for 48 to 72 h at 18°C for DNA extraction. The genomic DNA of *F. psychrophilum* was extracted using a Genomic DNA Isolation kit (QIAGEN, Hilden, Germany) as recommended by the manufacturer. Libraries were prepared for sequencing with the Nanopore sequencing kit SQK-NSK007 (Oxford Nanopore Technologies, Oxford, United Kingdom). The libraries were subsequently sequenced with the ONT MinION sequencer using rev C R9.4 flow cells (Oxford Nanopore Technologies, Oxford, United Kingdom) and sequencing runs were scheduled for 48–60 h. In general, 100-fold coverage was obtained for each genome. After sequencing, each read set was assembled individually with SPAdes version 3.8.1 and annotated with NCBI’s PGAAP pipeline.^1^

### Phylogenetic analysis of *Flavobacterium psychrophilum*

All *F. psychrophilum* genomes were available in GenBank metadata and included as of January 16, 2022. Our final datasets include 235 sequences generated for this study (list of GenBank accession numbers available in Supplementary Table S1). A phylogenetic tree of *F. psychrophilum* was inferred from SNPs identified by kSNP v 3.0 (Gardner et al., 2015) using a k-mer length of 19 nucleotides and a requirement that at least 75% of the genomes (i.e., 235 genomes) have a nucleotide at a given SNP position in order for the SNP to be considered to be core and included in tree building. A total of 521, 895 core SNP positions were identified. These SNPs were used to infer a maximum likelihood tree with RAxML v 8.2.X (Stamatakis, 2014) with 100 bootstrap replicates. The resulting tree was visualized by Figtree v1.4.3.^2^

### Phylogenetic analysis of Cas9 orthologs

The CRISPRCasMeta tool was used to retrieve Cas9 sequences from representative genomes. The multiple alignment was built using the MUSCLE program (Edgar, 2004). The FastTree program was used for the tree reconstruction. The visualization, annotation, and management of the phylogenomic tree were performed using Figtree v1.4.3.

### Phylogenetic analysis of genus *Flavobacterium*

We used PhyloPhlAn 3.0 (Asnicar et al., 2020) to generate the phylogeny of 484 *Flavobacterium* genome sequences including 196 species-identified strains and 288 unidentified strains available in the GenBank database as of January 20, 2022 (Supplementary Table S2). The visualization, annotation, and management of the phylogenomic tree were performed using Figtree v1.4.3.

### Bacterial RNA sequencing

*F*. *psychrophilum* CN46 was grown in 25 mL fresh TYES broth and incubated at 18°C with shaking at 120 rpm, until the OD_600_ reached 1.3. Bacterial cultures were then centrifuged (5,000 rpm) and resuspended in TRIzol (Thermo Fisher Scientific, 15,596,026). Total RNA was extracted with a RiboPure Bacteria kit (Thermo Fisher Scientific, AM1925). RNA was DNase I (Zymo research) treated and 3 dephosphorylated with T4 PNK (NEB, M0201). Ribo-Zero rRNA Removal Kit (Gram-Negative Bacteria) kit (Illumina, 15,066,012) was used to remove ribosomal RNA. The rRNA-depleted RNA was fragmented and reverse-transcribed to cDNA for library. The cDNA library was sequenced using an Illumina HiSeq X 10 platform with PE150 mode. Raw data were filtered by removing reads with adapters, reads with poly-N sequences, and reads of low-quality to obtain clean data.

### RNA sequencing analysis

The clean reads of RNA sequencing were aligned to the reference CN46 type II-C locus using BWA aligner and SAMtools (Li et al., 2009; Li and Durbin, 2009). Determined coordinates of 5′ and 3’ RNA ends were used to reconstruct the full-length RNA sequences. The resulting fragments were analyzed using IGV 2.1.4 (Thorvaldsdóttir et al., 2013).

### Conjugative transfer of plasmids into *Flavobacterium psychrophilum*

Plasmids were transferred from *E. coli* strain S17-1(λ-pir) into *F. psychrophilum* strain CN46 by conjugation as described earlier (Barbier et al., 2020). Briefly, *E. coli* strains carrying plasmids were incubated for overnight with shaking at 220 rpm in LB broth containing 100 μg/mL ampicillin at 37°C. *F*. *psychrophilum* strain CN46 was incubated for 72 h with shaking in TYES broth at 18°C. 900 μL *E. coli* cells were collected by centrifugation at 3,220 × g for 5 min and washed twice with 600 μL TYES. 900 μL *F. psychrophilum* cells were added to the bacterial precipitation, collected by centrifugation, and suspended in 400 μL TYES. The suspensions were spotted on TYES agar, and incubated at 18°C for 48 h. The cells were removed from the plate with a scraper and suspended in 400 μL TYES. 50 μL suspensions were spotted on TYES agar containing 10 μg erythromycin per mL and incubated at 18°C for 7 to 9 days.

### Plasmid interference assay

To construct an *E. coli* – *F. psychrophilum* shuttle plasmid, Primers CN101 (introducing a BamHI site) and CN102 (introducing a PstI site) were used to amplify a 2, 022-bp fragment using plasmid pCN06R1 as a template (Table 1). The resulting fragment was digested with BamHI and PstI and then ligated into the plasmid pLYL03, which had been digested with the same enzymes, to generate the pLC. Plasmid pLC was used as a template for adding a protospacer sequence followed by the putative PAM sequence 5’-NNAAAG-3′. Three interference plasmids were constructed carrying a protospacer which perfectly matched the spacer 2, spacer 3 and spacer 7 of *F. psychrophilum* strain CN46 followed by the natural PAM sequence in phage 1H or 2A, respectively. Furthermore, one control plasmid was constructed carrying a nonmatching control sequence followed by the natural PAM sequence located in phage 1H. These four plasmids were constructed by annealing of oligonucleotides CN201/CN202 (pLC-proto2), CN301/CN302 (pLC-proto3), CN401/CN402 (pLC-proto7), and CN501/CN502 (pLC-contr), followed by ligation in pLC cut by PstI and SphI (Table 2). The resulting plasmids were transformed chemically into competent *E. coli* strain S17-1 (λ-pir). Successful transformation and insertion were verified by colony PCR and Sanger sequencing.

**Table 1:** Key features of the three *Flavobacterium psychrophilum* genomes.

	CN06	CN38	CN46
Size, bp	2,836,981	2,830,982	2,826,602
G + C content, %	32.6	32.7	32.6
No. of CDS	5,497	5,454	5,454
rRNA, *n*	18	18	19
16S	6	6	6
23S	6	6	6
5S	6	6	7
tRNA, *n*	50	50	49
Plasmids, *n*	1	0	0
Prophages, *n*	4	1	1
IS elements, *n*	35	25	33
IS1595	1	1	0
IS982	8	7	7
IS256	12	9	14
ISFps1	2	1	0
ISAS1	2	1	2
IS30	2	1	2
IS1182	3	3	0
IS3	8	2	5
IS200	0	0	1
IS630	0	0	2

CDS, coding sequences; IS, insertion sequence. Data refer to the chromosome only, except where plasmids were indicated.

**Table 2:** Bacterial strains, plasmids, and primers used in this study.

Plasmid, strain, primer	Description/Sequence (5′- > 3′)^α,β^	Source or references
pLYL03	ColE1 ori; Ap^r^ (Em^r^)	McBride and Baker (1996)
pCN06R1	A wild-type plasmid of *F. psychrophilum* strain CN06	This study
pLC	*E. coli* – *F. psychrophilum* shuttle plasmid; ColE1 ori (pCN06R1 ori), Ap^r^ (Em^r^)	This study
pLC-proto2	pLC-derivative plasmid carrying a protospacer that perfectly match the spacer 2 of CN46 followed by a PAM motif; Ap^r^ (Em^r^)	This study
pLC-proto3	pLC-derivative plasmid carrying a protospacer that perfectly match the spacer 3 of CN46 followed by a PAM motif; Ap^r^ (Em^r^)	This study
pLC-proto7	pLC-derivative plasmid carrying a protospacer that perfectly match the spacer 4 of CN46 followed by a PAM motif; Ap^r^ (Em^r^)	This study
pLC-contr	pLC-derivative plasmid carrying a nonmatching sequence followed by a PAM motif; Ap^r^ (Em^r^)	This study
Strains		
*F. psychrophilum* strains		
CN06	*F. psychrophilum* isolated from *Oncorhynchus mykiss*	This study
CN38	*F. psychrophilum* isolated from *Oncorhynchus mykiss*	This study
CN46	*F. psychrophilum* isolated from *Thymallus thymallus*	This study
CN46-pLC	CN46 carrying plasmid pLC; Apr (Em^r^) (Em^r^)	This study
CN46-proto2	CN46 carrying plasmid pLC-proto2; Ap^r^ (Em^r^)	This study
CN46-proto3	CN46 carrying plasmid pLC-proto3; Ap^r^ (Em^r^)	This study
CN46-proto7	CN46 carrying plasmid pLC-proto7; Ap^r^ (Em^r^)	This study
CN46-contr	CN46 carrying plasmid pLC-contr; Ap^r^ (Em^r^)	This study
*E. coli* strains		
S17-1 (λ-pir)	Strain used for conjugation	Li et al. (2017)
Primers		
CN101	ATCAGGATCCAGCAATAGAAACCTCCAATT	
CN102	ATCGCTGCAGCCTGGTTTACTTTCAATCTT	
CN201	ACCAATTTTTGATACATCGTATTAAATCGC**CAAAAG**CATG	
CN202	ACGTCTTTTGGCGATTTAATACGATGTATCAAAAATTGGT	
CN301	CGTCTATAAAATCATTACTAGAAGCTCTTA**CTAAAG**CATG	
CN302	ACGTCTTTAGTAAGAGCTTCTAGTAATGATTTTATAGACG	
CN401	TTGCATCTTTTTTTTGTTTTTACGAAGATA**CAAAAG**CATG	
CN402	ACGTCTTTTGTATCTTCGTAAAAACAAAAAAAAGATGCAA	
CN501	TTTGTTCTTTTGTATAATTTACATTTTCTC**CAAAAG**CATG	
CN502	ACGTCTTTTGGAGAAAATGTAAATTATACAAAAGAACAAA	

^α^Antibiotic resistance phenotypes: Ap^r^, ampicillin; Em^r^, erythromycin. Unless indicated otherwise, the antibiotic resistance phenotypes were those expressed in *E. coli*. The antibiotic resistance phenotypes given in parentheses was those expressed in *F. psychrophilum* but not in *E. coli*. ^β^The restriction sites were underlined and the PAM motif sequences downstream of protospacers were in bold.

In the conjugation assay, the interference plasmids were transferred into *F. psychrophilum* strain CN46. Both the donor and the recipient cells were transferred to fresh medium (LB for *E. coli* and TYES medium for *F. psychrophilum*) from a freezer stock. *E. coli* was grown to an OD_600_ of 0.80, while the recipient *F. psychrophilum* strain was grown to an OD_600_ of 0.90. The conjugation protocol was performed similarly to the conjugative transfer of plasmid into *F. psychrophilum* described above, and the final donor/recipient mixtures were spotted on TYES agar plates. After 48 h of growth at 18°C, the cells were scraped and resuspended in 0.4 mL TYES broth and 50 μL of the mixed cultures were spotted on TYES agar plates containing 10 μg erythromycin per mL. Colonies were photographed after 7–9 days at 18°C. The conjugation assay was repeated three times.

## Results

### Comparative genomics identifies a gene encoding a novel endonuclease Cas9 in *Flavobacterium psychrophilum*

Recently, we have revealed that important epidemiological and ecological aspects of 31 *F. psychrophilum* strains isolated from four provinces in China (Li et al., 2021). In this work, we demonstrated that 7 isolates belonged to ST 12 whereas 23 isolates belonged to three novel sequence types (STs) including ST355, ST356, and ST357. These results indicated *F. psychrophilum* isolates from China existed genetic diversity. However, the genomic diversity and evolution of *F. psychrophilum* isolates from China remain unclear. In this work, we performed Whole Genome Sequencing (WGS) of CN06, CN38, and CN46 to understand the genetic diversity in *F. psychrophilum* isolates in China. The genome sizes of CN06, CN38, and CN46 ranged from 2.83 to 2.84 Mbp (Table 1), similar in length to other completely sequenced *F. psychrophilum* genomes (range from 2.72 to 2.90 Mbp) (Duchaud et al., 2018), and exhibited an average nucleotide identity (ANI) of >99.4% to each other. The ANI to the type strain OSU THCO2-90 was around 99.46% [99.45–99.49%], and the *in silico* DNA–DNA hybridization (isDDH) values were around 86.33% [85.1–87.1%], indicating that they were indeed *bona fide* members of the species *F. psychrophilum*. *In silico* multilocus sequence typing analysis of seven target genes (*atpA*, *dnak*, *fumC*, *gyrB*, *murG*, *trpB*, and *tuf*) developed by Nicolas et al. (2008) revealed that CN06 was a member of ST12 and CC10 whereas CN38 and CN46 were members of a hitherto unidentified sequence type and clonal complex. CN06 harbored four prophages and one wild type plasmid called pCN06R1 whereas CN38 and CN46 carried only one prophage (Table 1), indicating that these *F. psychrophilum* strains were constantly challenged by invading genetic elements in natural environments. A highly unusual feature of these three genomes was the presence of a large number of transposase genes (Table 1). Strikingly, CN06 harbored eight IS982 transposases whereas CN38 and CN46 harbored seven IS982 transposases, respectively. Furthermore, CN06 harbored twelve IS256 transposases whereas CN38 and CN46 harbored nine and fourteen IS256 transposases, respectively. This data showed that both IS982 and IS256 transposases existed widely in *F. psychrophilum* strains.

**Figure 1:**
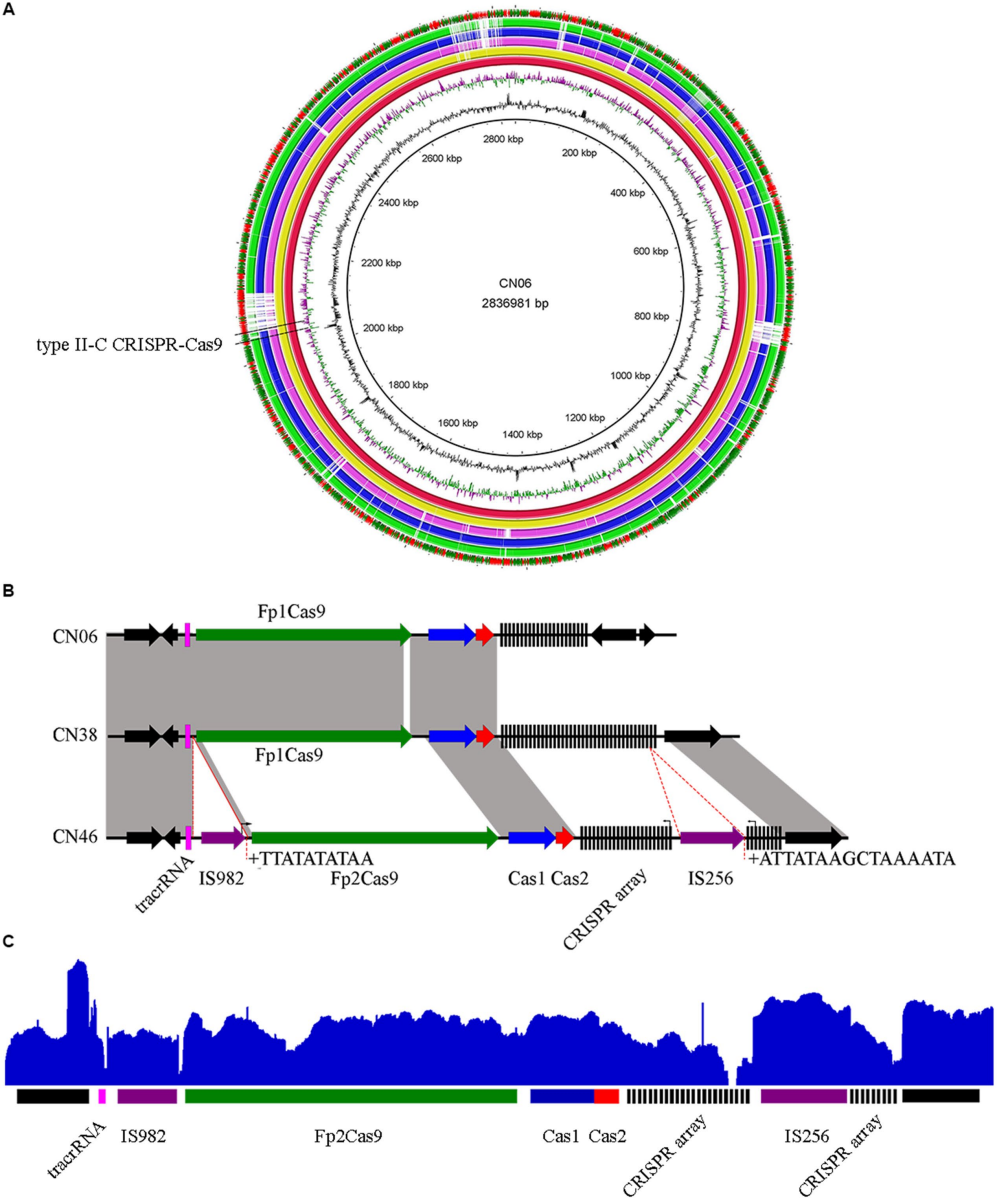
**(A)** The locations of type II-C CRISPR-Cas9 systems in *F. psychrophilum* isolates. Three closed genomes of *F. psychrophilum* isolates (CN06, CN38, and CN46) were compared with two representative *F. psychrophilum* genomes (OSU THCO2-90 and JIP02/86). Genes that are present in CN06 (red inner circle) but not in the other genomes were shown as blank spaces in the rings representing the 4 genomes. The figure was constructed using BRIG v0.95. **(B)** Comparison of genomic organization of the FpCas9 genes neighborhood in isolates CN06, CN38, and CN46. Direct repeats (DRs) were shown as black rectangles while spacers were indicated by short black lines. The tracrRNA coding sequences were shown as pink rectangles. The *cas1*, *cas2*, *cas9* genes were shown as blue, red, and green arrows, respectively. Transposases were shown as purple arrows. Vertical arrows indicated putative motifs corresponding to the consensus sequence of *Bacteroidetes* promoters (TAnnTTTG) or repeat-encoded promoters (TTG). Target site duplications at insertion sites of IS982 and IS256 were labeled as +. No significant rho-independent terminator was identified using the ARNold software for finding terminators (http://rna.igmors.u-psud.fr/toolbox/arnold). **(C)** Mapping of mRNA reads revealed by RNA-seq was shown at the top of the CN46 CRISPR-Cas type II-C locus in blue.

Comparison of the three genome contents revealed that each of them harbored an intact type II-C CRISPR-Cas9 system located in the same region of *F. psychrophilum* genomes (Figure 1A), which comprised tracrRNA, endonuclease Cas9 called FpCas9, adaptation proteins Cas1 and Cas2, and a CRISPR array (Figure 1B). Comparison of these type II-C Loci revealed a novel FpCas9 called Fp2Cas9 that was present in isolate CN46 but not in isolates CN06 and CN38 (Figure 1B; Supplementary Figure S1). Compared with isolate CN38 but not CN06, Fp2Cas9 was encompassed in a set of orthologous genes displaying a conserved organization and located between highly conserved genome genes (Figure 1B). These results suggested that FpCas9 genetic exchange occurred through homologous recombination between isolate CN38 and isolate CN46. A highly unusual feature of the type II-C locus in isolate CN46 was the presence of two new inserted transposase genes encoding an IS982 transposase between tracrRNA and Fp2Cas9 gene and an IS256 transposase inside the CRISPR array (Figure 1B). To determine the transcriptional structure of the type II-C locus, mRNAs present in CN46 were sequenced. The results showed that both two truncated parts of the CRISPR array and the *cas* genes were actively transcribed (Figure 1C). Previous works had shown that *F. columnare* type II-C CRISPR array was probably expressed within the array toward the variable end using repeat-encoded promoter sequence (5′-TTG-3′) (Hoikkala et al., 2021). The *F. psychrophilum* type II-C repeats had the same motif 35 bp upstream of each spacer, indicating that this putative promoter also drove the II-C CRISPR array transcription in *F. psychrophilum*. A motif sequence corresponding to the *Bacteroidetes*-specific promoter sequence TAnnTTTG was also identified for Fp2Cas9 gene (Rochat et al., 2019), indicating that the transcription might be initiated 153 bp upstream of its start codon. This putative promoter was located in the coding region of the IS982 transposase (Figure 1B). Because no Rho-independent terminator was identified in the IS982-Fp2Cas9, Fp2Cas9-Cas1, and Cas1-Cas2 intergenic region, both *cas1* and *cas2* genes could also cotranscribed with Fp2Cas9 gene.

### Fp2Cas9 is a newly acquired endonuclease Cas9 in *Flavobacterium psychrophilum*

**Figure 2:**
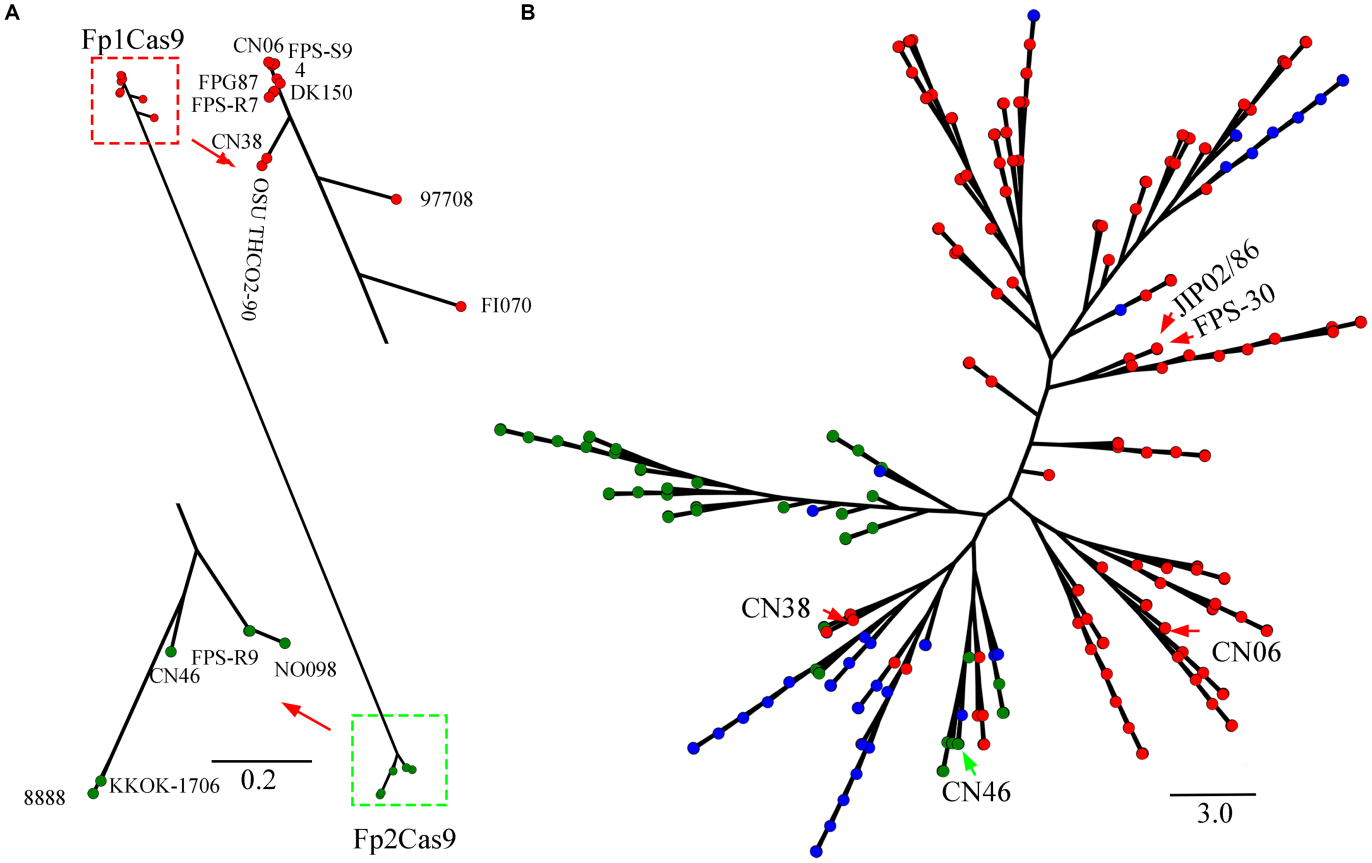
**(A)** Evolution of FpCas9 orthologs. Cas9 protein sequences were manually retrieved from 235 genomes. The multiple alignments for the representative set of intact Cas9 sequences were constructed using the MUSCLE program. The FastTree program was used for the FpCas9 tree reconstruction. Fp1Cas9 and Fp1Cas9 variants were noted as red circles while Fp2Cas9 and Fp2Cas9 variants were noted as green circles. **(B)** Phylogenetic networks of *Flavobacterium psychrophilum*. A phylogenomic tree based on the comparative analysis of the core SNPs derived from the genomes of 235 *F. psychrophilum* strains including CN06, CN38, and CN46. The maximum likelihood phylogeny was inferred using kSNP and RAxML (with 100 bootstrap values) based on the concatenated core SNPs (*n* = 4,457). The strains that harbored an intact Fp1Cas9, Fp1Cas9 variants or Fp1Cas9 fragments were noted as red circles, the strains that harbored an intact Fp2Cas9 or Fp2Cas9 variants were noted as green circles, and the strains that did not harbor any FpCas9 were noted as blue circles.

To confirm the existence of Fp2Cas9 in *F. psychrophilum*, the relationship of Fp2Cas9 from isolate CN46 was compared with intact FpCas9 orthologs in the genomes of a representative set of 234 strains covering all of the currently sequenced *F. psychrophilum* strains (Supplementary Table S1). Phylogenetic analysis distributed these FpCas9 orthologs into two major clades (Figure 2A). The majority of *F. psychrophilum* strains including JIP02/86, FPS-30, and CN06 harbored an Fp1Cas9. Phylogenetic analysis also showed that 10 strains including FPS-R7, CN38, DK150, 97,708 and FI070 harbored different Fp1Cas9 variants (Figure 2A). Compared with Fp1Cas9, we further demonstrated that the C-terminus of these Fp1Cas9 variants was mainly variable (Supplementary Figure S2). Phylogenetic analysis showed that eight strains including 96,233, CN46, and DK095 harbored the endonuclease Fp2Cas9 (Figure 2A). Phylogenetic analysis also showed that several strains including 8,888, FPS-R9 and NO098 harbored different Fp2Cas9 variants (Figure 2A). We further demonstrated that the C-terminus of these Fp2Cas9 variants was mainly variable compared with Fp2Cas9 (Supplementary Figure S3). This data showed that two different FpCas9 orthologs coexisted in *F. psychrophilum* strains and these two FpCas9 orthologs had evolved to different variants in natural environments.

To further gain a better understanding of the emergence of Fp2Cas9, the phylogenomic relationship of CN46 was compared with the genomes of these above *F. psychrophilum* strains. Phylogenomic analysis distributed these 235 strains into eight lineages (Figure 2B). In the resulting tree, these strains evolved from a common ancestor that was closely related to JIP02/86 and FPS-30. Given the presence of an intact Fp1Cas9 in JIP02/86 and FPS-30 genomes, Fp1Cas9 emerged in *F. psychrophilum* before earlier than Fp2Cas9. Furthermore, CN38 and CN46 clustered into the same clade, in which both Fp1Cas9 and Fp2Cas9 coexisted (Figure 2B). This data showed that CN38 and CN46 were closely related and suggested they originated from a recent common ancestor. Based on phylogenetic analysis described above, it appears that Fp2Cas9 in strain CN46 was acquired through horizontal gene transfer from other bacteria and then integrated into the type II-C locus of strain CN38 instead of Fp1Cas9.

### Fp2Cas9 originated from a *Flavobacterium* species

**Figure 3:**
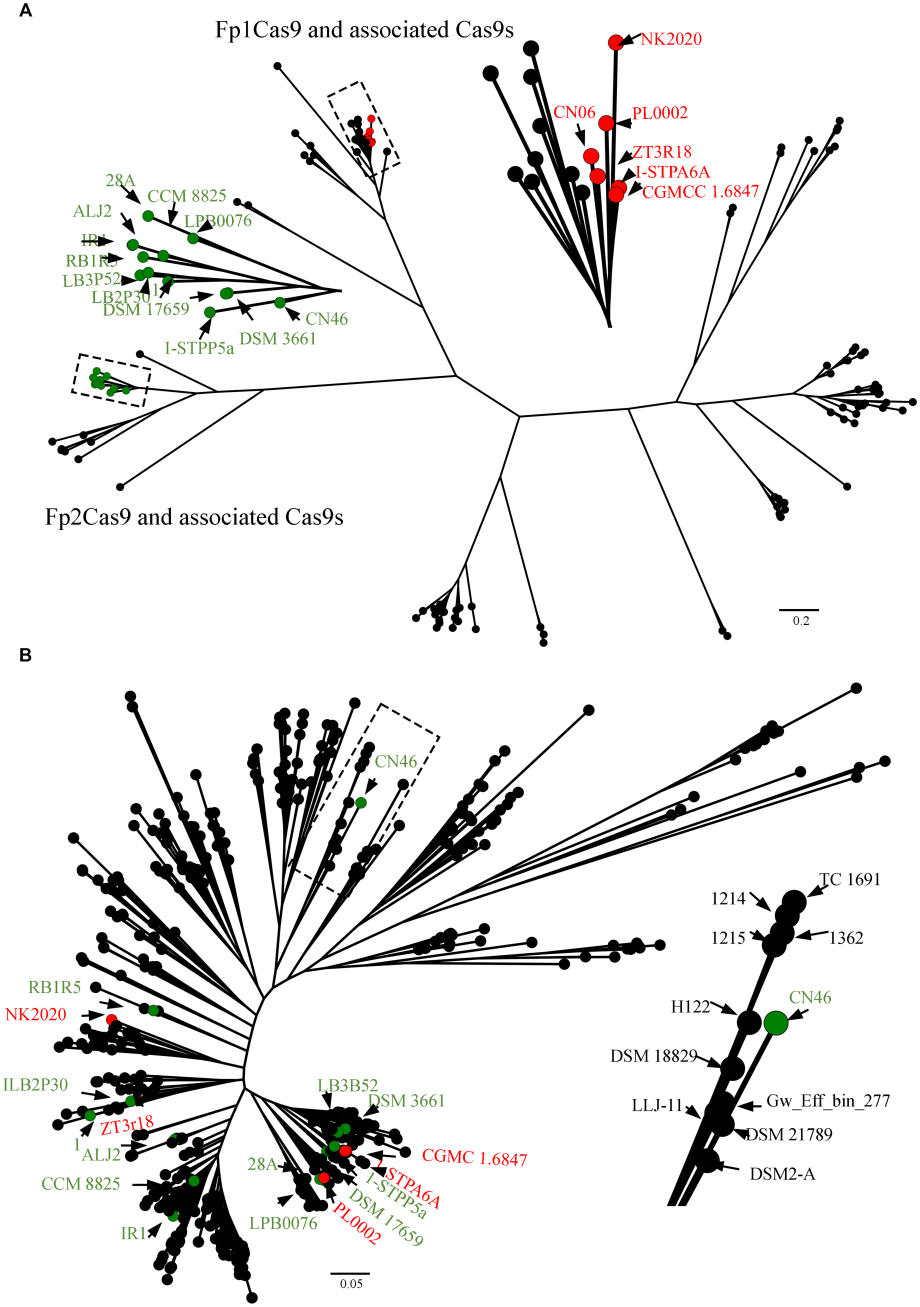
**(A)** Origin of *F. psychrophilum* Fp2Cas9. The CRISPRCasMeta tool was used to retrieve Cas9 protein sequences from 483 genomes in the GenBank database. The multiple alignments were built using the MUSCLE program and the FastTree program was used for the tree reconstruction. **(B)** Phylogenetic network of the relationships between species in the genus *Flavobacterium*. The phylogenomic tree was constructed using PhyloPhlAn 3.0 which used 400 optimized universal marker sequences to produce a panmicrobial phylogeny. The visualization, annotation, and management of the phylogenomic tree were performed using FigTree. Fp1Cas9 and associated Cas9 orthologs were noted as red circles whereas Fp2Cas9 and associated Cas9 orthologs were noted as green circles.

To confidently predict the evolutionary origin of *F. psychrophilum* Fp2Cas9, all 483 genomes of *Flavobacterium* species publicly available in GenBank database were selected to be analyzed for the presence of Cas9 orthologs with the use of the CRISPRCasMeta tool (Supplementary Table S2). Of these, 128 were predicted to harbor an intact Cas9 orthologs. We generated a representative set of these Cas9 sequences and built a phylogenetic tree from a multiple alignment of these Cas9 sequences (Figure 3A). The resulting tree strongly supported that Fp1Cas9 formed a distinct clade that consisted of Cas9 orthologs from *F*. sp. ZT3R18, *F*. *sp.* PL0002, *F*. *sp.* NK2020, *F. tiangeerense* CGMCC 1.6847 and *F*. *sp.* I-STPA6A, suggesting that these Cas9 orthologs were originated from a recent common ancestor. However, Fp2Cas9 formed a distinct clade that consisted of Cas9 orthologs from *F. xanthum* DSM 3661, *F*. *crassostreae* LPB0076, *F*. *psychrotolerans* RB1R5, *F*. *cupreum* CCM 8825, *F*. *franklandianum* LB3P52, *F*. *laiguense* LB2P30, *F. micromati* DSM 17659, *F*. *sp.* IR1, *F*. *sp.* 1, *F*. *sp.* I-STPP5a, *F*. *sp.* 28A, and *F*. *sp.* ALJ2 (Figure 3A). We further analyzed the nucleotide sequence similarity between Fp2Cas9 and its associated Cas9 orthologs. The results showed that Fp2Cas9 shared 89.46% nucleotide identity with a query cover of 87% with the Cas9 ortholog from *F*. *sp.* I-STPP5a, suggesting that isolate CN46 acquired Fp2Cas9 from an unidentified *Flavobacterium* species. To further demonstrate that *F. psychrophilum* acquired Fp2Cas9 by the horizontal gene transfer, the CN46 genome was selected to be compared with 483 genomes of *Flavobacterium* species using PhyloPhlAn 3.0 (Supplementary Table S2). The phylogenetic tree obtained with these sequences globally did not fit the tree obtained with Fp2Cas9-associated Cas9 sequences, suggesting horizontal gene transfer occurred frequently between *Flavobacterium* species (Figure 3B). The strain CN46 formed a closely related clade with these strains of *F*. *davisii* 1,215, *F*. *oreochromis* 1,214, *F. columnare* TC 1691, *F*. *covae* 1,362, *F*. *sp.* H122, *F. terrae* DSM 18829, *F*. *amnicola* LLJ-11, *F*. *sp.* Gw_Eff_bin_277, *F. swingsii* DSM 21789, and *F*. *humi* DS2-A (Figure 3B). However, CN46 was phylogenetically distant from those strains harboring Fp2Cas9-associated Cas9 orthologs (Figure 3B). These results suggested that Fp2Cas9 could be transmitted horizontally to *F. psychrophilum* from a *Flavobacterium* species.

### Fp2Cas9 provides adaptive interference against phage genomes

**Figure 4:**
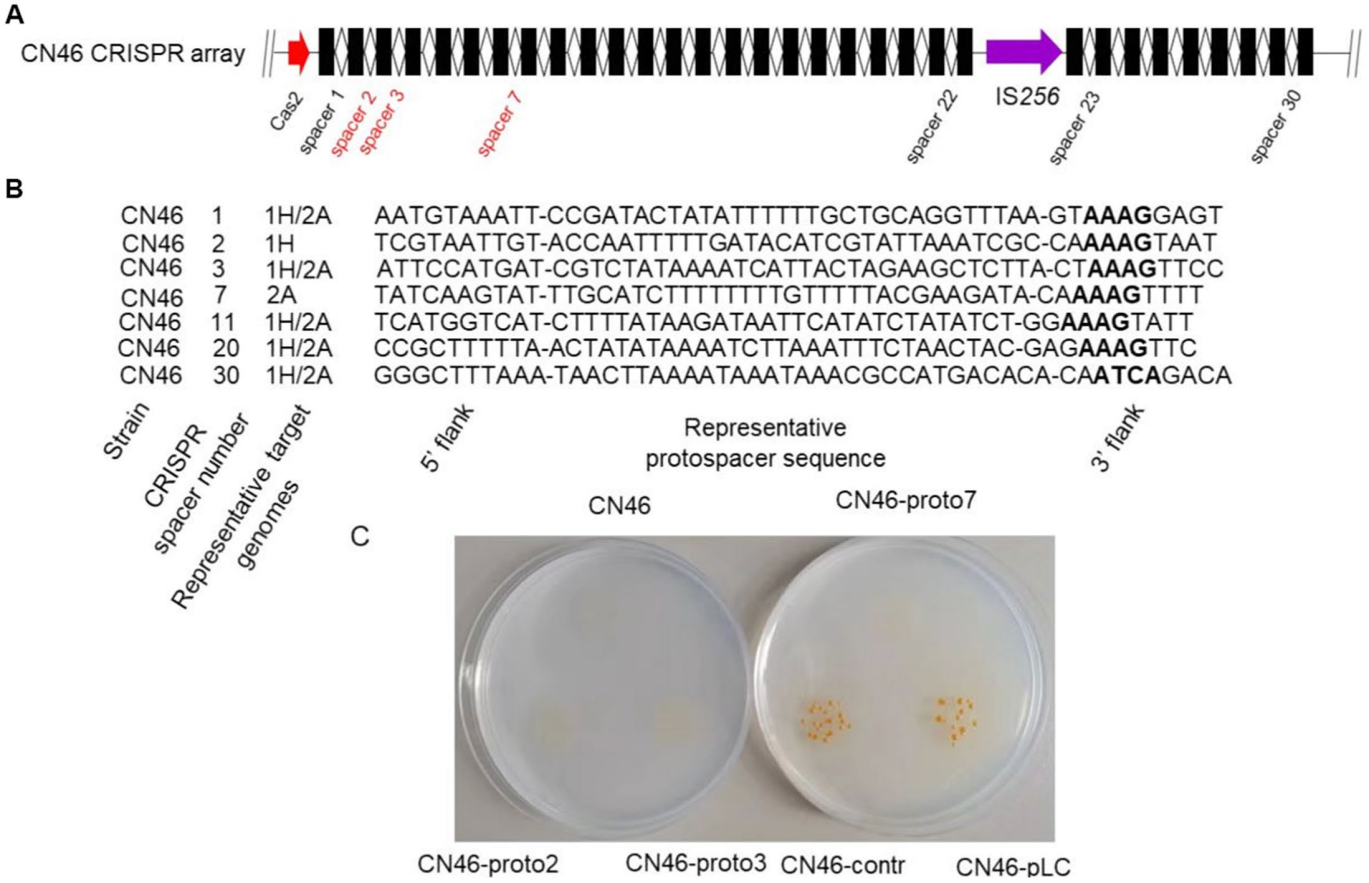
**(A)** Schematic representation of the CRISPR array from *F. psychrophilum* strain CN46. Repeats and spacers were shown as rectangles and diamonds, respectively. **(B)** Potential natural targets for the seven spacers in the CRISPR array from *F. psychrophilum* strain CN46. For each spacer, its number in the CRISPR arrays and representative target genomes were listed. The PAM regions in the putative targets were in bold. **(C)** *F. psychrophilum* conjugants on antibiotic agar plates. Cells were conjugated with either an interference plasmid carrying a protospacer, which perfectly matched the spacer 2, spacer 3, or spacer 7 of CN46, respectively, or a control plasmid carrying a nonmatching control sequence.

Having defined unique features and evolution of Fp2Cas9 from *F. psychrophilum* isolate CN46, we turned our attention toward the interference function of this endonuclease. The CRISPR array immediately downstream of *cas2* in isolate CN46 genome was analyzed (Figure 4A). The CRISPR array was divided into two parts by IS256 transposase and comprised 32 46 bp direct repeats (DRs) interspaced by 30 spacers. To get some insights on the potential origin of the spacers present in the CRISPR array, all 30 unique spacer sequences were retrieved and predicted for potential protospacers using CRISPRTarget program. Most spacers did not have any hits in the databases, whereas at least one potential target for 7 of them was identified (Figure 4B). Protospacer alignments revealed an apparent PAM motif of 5’-NNAAAG-3′ for Fp2Cas9. To confirm that Fp2Cas9 yield functional interference against *Flavobacterium* phages 1H and 2A, we performed a plasmid interference assay. We constructed an *E. coli*-*F. psychrophilum* shuttle plasmid called pLC and modified it to carry protospacers that perfectly matched the spacer 2, spacer 3, and spacer 7 in the CRISPR array from strain CN46, respectively. As a control plasmid, we replaced these matching protospacer sequences with a nonmatching sequence located in the phage 1H genome. Using *Escherichia coli* as the donor, CN46 was unable to receive plasmids carrying protospacers (Figure 4C). These results suggested that CRISPR interference in *F. psychrophilum* strain CN46 was effective against phage genomes.

## Discussion

Type II endonuclease Cas9 provides an adaptive interference against multiple invading genetic elements, such as bacteriophages and plasmids. Previous works reported that several *F. psychrophilum* strains harbored a type II-C endonuclease called Fp1Cas9 (Castillo et al., 2015). Despite many attempts, this endonuclease has not been well characterized to date and failed to provide adaptive interference against invading genetic elements (Castillo et al., 2015; Daniel et al., 2021; Jørgensen et al., 2022). In this work, we took advantage of the availability of the genome sequences of 3 *F. psychrophilum* isolates, namely, CN06, CN38, and CN46. We identified a novel type II-C endonuclease Cas9 called Fp2Cas9 that was present exclusively in *F. psychrophilum* isolate CN46. We revealed that *F. psychrophilum* acquired a novel endonuclease Cas9 from the CRISPR-Cas locus in another *Flavobacterium* species which was then integrated into the previous type II-C locus and the transcription of this endonuclease was driven by a newly integrated promoter sequence. This integrated Fp2Cas9 provides adaptive interference against foreign plasmids. Future studies would be necessary to determine the adaptation function of spacers against bacteriophage infections.

Based on the phylogenetic analysis of FpCas9 orthologs in 235 *F. psychrophilum* isolates, we found that two different type II-C FpCas9 orthologs, namely Fp1Cas9 and Fp2Cas9, existed in *F. psychrophilum* isolates (Supplementary Table S1). We further demonstrated that some strains harbored truncated Fp1Cas9 (e.g., FPG3) while some strains lacked any of these endonucleases (e.g., 950,106-1/1). This could reflect an evolutionary history of FpCas9 loss or acquisition in *F. psychrophilum* strains. Although Fp2Cas9 had a higher amino acid similarity with *F*. *cloumnare* type II-C Cas9 (FcCas9) than Fp1Cas9, Fp2Cas9 and FcCas9 recognized different PAM sequences (Supplementary Figure S1) (Hoikkala et al., 2021). Given that Cas9 can participate in the selection of PAM sequences during spacer acquisition and adaptive interference, it appears that these two type II-C FpCas9s recognize different PAMs. The PAM-interacting domain, which is usually located in the C-terminus of Cas9 nucleases and directly interacted with PAM sequence, is widely considered to be the major cause of PAM specificity (Nishimasu et al., 2015; Anders et al., 2016; Xie et al., 2022). We found that the C-terminus of these two FpCas9s was variable in several *F. psychrophilum* strains (Supplementary Figures S2, S3). This finding suggested that these FpCas9 variants might recognize different PAMs. Thus, it seems that *F. psychrophilum* isolates have evolved to acquire novel nuclease Cas9 or change the PAM-interacting domain of FpCas9 to expand PAM recognition against invading genetic elements. Future studies are needed to reveal the interference mechanisms of FpCas9s and their variants against bacteriophage infections.

Given that Fp2Cas9 existed in *F. psychrophilum* isolates later than Fp1Cas9 and all Fp2Cas9-homologous proteins were members of the genus *Flavobacterium*, it suggested that Fp2Cas9 was a newly acquired endonuclease from a *Flavobacterium* species. Strain CN46 had a relatively closer relationship with strain CN38 than strain CN06. Importantly, the location of FpCas9 gene was conserved which was located between tracrRNA and *cas1* in the genomes of both CN38 and CN46 and the neighboring regions are highly conserved, the Fp2Cas9 gene was likely acquired from a *Flavobacterium* species, and then integrated into the type II-C locus of CN38, resulting a Fp2Cas9-carrying *F. psychrophilum* strain. Because there were lots of IS982 and IS256 transposase genes in CN38 genome, it might be suggested that these transposase genes might be very successful in multiplying across the genome of CN38. The IS982 transposase gene was inserted into the tracrRNA-FpCas9 region whereas the IS256 transposase gene was inserted into the CRISPR array in the Fp2Cas9-carrying *F. psychrophilum* strain, resulting in the type II-C locus of strain CN46.

Previous study indicated that the type II-C locus from *F. columnare* strain B245 provided limited interference efficiency against target plasmids (Hoikkala et al., 2021). Our conjugation assay showed that the type II-C CRISPR system from strain CN46 provided completely adaptive interference against target DNA plasmids, suggesting that the nucleotide sequence 5’-NNAAAG-3′ (where N was any nucleotide) was probably one of PAMs recognized by Fp2Cas9 and Fp2Cas9 could efficiently cleave protospacers in target plasmids with crRNA and tracrRNA. Due to the conjugation efficiency for strain CN46 was very low and subjected to many factors such as restriction modification systems (Alvarez et al., 2004), it did not seem to be the most efficient way to access the PAM preference and Fp2Cas9 endonuclease activity. Fedorova et al. found that type II-C CRISPR systems could be transformed to a bacteria model lacking related CRISPR-Cas9 system such as *E. coli* strains to confirm the interference function of type II-C Cas9s (Fedorova et al., 2020a,b). However, we could not detect the intact expression of Fp2Cas9 in *E. coli* strains, such as BL21(DE3) and Rosetta (DE) plysS, which maybe due to the existence of rare codons in Fp2Cas9 (data not shown here). Future studies are needed to demonstrate the interference function of this novel type II-C CRISPR-Cas9 system using codon-optimized Fp2Cas9 in *E. coli* strains or another bacterial model in which the type II-C system works efficiently.

Using bacterial RNA-seq experiment, the result showed that the CRISPR arrays truncated by IS256 were actively transcribed into pre-crRNAs while the Fp2Cas9 gene was efficiently transcribed. Bioinformatics analysis showed that the type II-C CRISPR arrays were likely transcribed within the arrays toward the variable end using repeat-encoded promoter sequence. This transcription way was observed in type II-C CRISPR arrays from several bacteria (Zhang et al., 2013; Hoikkala et al., 2021). In *F. columnare*, a related bacterium with *F. psychrophilum,* the type II-C CRISPR array was efficiently driven by the same repeat-encoded promoter sequence (Hoikkala et al., 2021). Bioinformatics analysis also showed that the Fp2Cas9 gene was likely transcribed using a *Bacteroidetes*-specific promoter sequence located in the IS982 transposase gene. Recently, this transcription way was observed in *F. psychrophilum* strains (Rochat et al., 2019). However, we did not reveal the transcription mechanism of tracrRNA and the maturation mechanisms of crRNA and tracrRNA. Future studies would be necessary to determine the transcription mechanism of tracrRNA and the maturation mechanisms of crRNA and tracrRNA.

**Figure 5:**
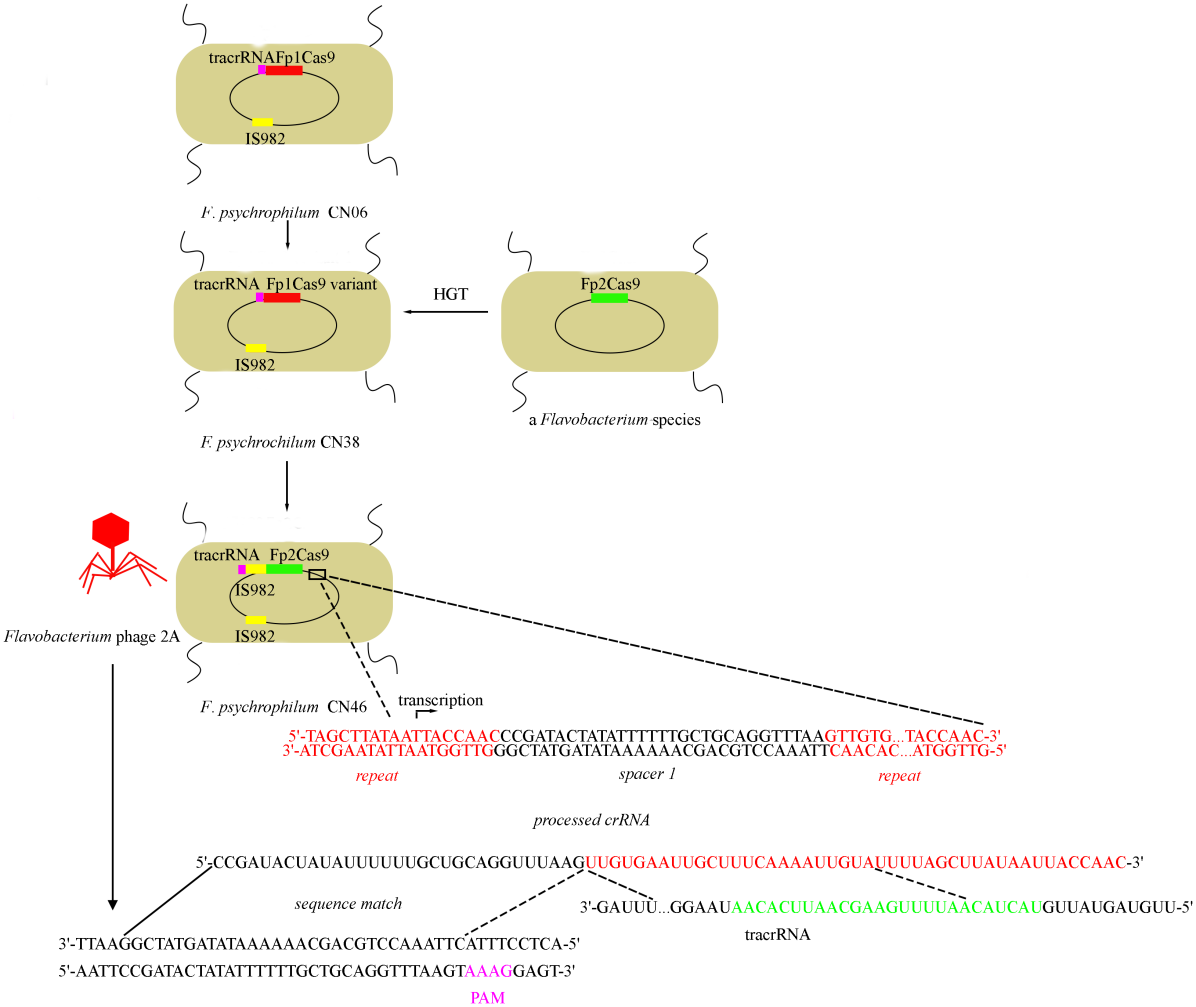
Model for Fp2Cas9 evolution and interference mechanism in *F. psychrophilum*. *F. psychrophilum* isolates were proposed to originate from a common ancestor harboring an Fp1Cas9 gene. Subsequent recombination events led to replacement of the Fp1Cas9 gene with the Fp2Cas9 gene from a *Flavobacterium* species. And then, IS982 transposase was integrated into the tracrRNA-Fp2Cas9 region and the transcription of Fp2Cas9 was driven by a promoter sequence located in the IS982 transposase. Mapping the crRNA onto the *Flavobacterium* phage genomes revealed a downstream PAM (pink letters on the *Flavobacterium* phage 2A genome) which was confirmed by plasmid interference assay. All sequences shown here corresponded to actual genomic sequences.

Based on our evolutionary analysis and biochemical studies, we propose a model for *F. psychrophilum* Fp2Cas9 evolution and interference function (Figure 5). Fp1Cas9 from strain CN06 was mutated to Fp1Cas9 variant from strain CN38. Fp2Cas9 from strain CN46 was acquired through horizontal gene transfer from a *Flavobacterium* species and then integrated into the type II-C locus from CN38 instead of the Fp1Cas9 variant. IS982 transposase was then integrated into the tracrRNA-Fp2Cas9 region and drove the transcription of Fp2Cas9. The CRISPR array was transcribed into pre-crRNAs driven by repeat-encoded promoter sequence which was further matured into crRNA. tracrRNA was transcribed and matured. crRNA bound to the processing tracrRNA-Cas9 complex and then served as a guide to recognize protospacers in *Flavobacterium* phage genomes. Fp2Cas9 recognized the PAM sequences and cleaved the target DNA sequences, resulting in resistance against phage infections.

In this work, we revealed the origin and evolution of a novel type II-C CRISPR-Cas9 system from the fish pathogen *F. psychrophilum* for the first time. Future studies will focus on the adaptive interference function of this novel endonuclease.

## Data availability statement

The datasets presented in this study can be found in online repositories. The names of the repository/repositories and accession number(s) can be found in the article/Supplementary material.

## Ethics statement

The animal study was reviewed and approved by The Committee of the Ethics on Animal Care and Experiments at Heilongjiang River Fisheries Research Institute of Chinese Academy of Fishery Sciences.

## Author contributions

FC: conceptualization, data curation, formal analysis, funding acquisition, investigation, methodology, project administration, resources, software, supervision, validation, visualization, and writing – original draft. DW: conceptualization, methodology, project administration, and writing – review and editing. TL: funding acquisition, supervision, and writing – review and editing. SL: conceptualization, methodology, project administration, supervision, writing – original draft, and writing – review and editing. All authors contributed to the article and approved the submitted version.

## Funding

This work was financially supported by Central Public-interest Scientific Institution Basal Research Fund, HRFRI (no. HSY202001M) and Central Public-Interest Scientific Institution Basal Research Fund, CAFS (no. 2020TD43).

## Conflict of interest

The authors declare that the research was conducted in the absence of any commercial or financial relationships that could be construed as a potential conflict of interest.

## Publisher’s note

All claims expressed in this article are solely those of the authors and do not necessarily represent those of their affiliated organizations, or those of the publisher, the editors and the reviewers. Any product that may be evaluated in this article, or claim that may be made by its manufacturer, is not guaranteed or endorsed by the publisher.

## Footnotes

^1^ http://www.ncbi.nlm.nih.gov/genome/annotation_prok/

^2^ http://tree.bio.ed.ac.uk/software/figtree/
