# Supplementary material for: Identification of a novel type II-C Cas9 from the fish pathogen Flavobacterium psychrophilum
Source: Front Microbiol. 2023 Jun 15;14:1181303. doi: 10.3389/fmicb.2023.1181303 (PMC10309648; doi:10.3389/fmicb.2023.1181303)
Supplement: Supplementary file 3 [file Data_Sheet_1.PDF]

Fig. S1 FpCas9(CN6), FpCas9(CN38), FpCas9(CN46) and *Flavobacterium columnare* Cas9 (FcCas9) protein alignment.

```
FpCas9(CN6)_  MKKILGLDLGTNSIGWAFINEAENEIEVSSIVNTGVRIVPLTTDEESDFKKG.NTISINA
FpCas9(CN38)  MKKILGLDLGTNSIGWAFINEAENEIEVSSIVNTGVRIVPLTTDEESDFKKG.NTISINA
FpCas9(CN46)  MKKILGLDLGTNSIGWALTNNDF.DNKLGNIEGLGSRIIPMSQDVLGKFDSG.VSISQTA
FcCas9        MKFNLGLDIGTNSIGWALVNEDY.ENKKGKIIAAGSRIIPMTQDILDKFGSGAITETQTA

FpCas9(CN6)   DRTLKRGARRGLQRFKQRREALLETFNRIKFIATDFVYAETGAATTFSSYQLRAKSAVEA
FpCas9(CN38)  DRTLKRGARRGLQRFKQRREALLEIFSRIKFIATDFVYAETGASTTFSSYQLRAKSAVEA
FpCas9(CN46)  ERTKYRGTRRLVQRFLLRRERLHRILNILDFLPKHYAETID.FEKKFGQFKPEKEEKLSF
FcCas9        LRTDYRSKRKLIQRFLLRRERLHRVLNVLDFLPKHYAESID.FEKYFGKFLPNTEPKLAY

FpCas9(CN6)   VTKEELVKVLLMLNKKRGYKSSRKAKTAEEGDAIDGMKIAKEIFENNLTPGQWVYNSLSK
FpCas9(CN38)  VTKEELVKVLLMLNKKRGYKSSRKAKTAEEGDAIDGMKIAKEIFENNLTPGQWVYNSLSK
FpCas9(CN46)  YKNESGKNQFLFLES...HQEMLAEFQLINPDL....KQVPYDWTIYYLRKKALTQKISK
FcCas9        NQN.....QFIFQNS...FLEMLKEFQEKHPELLTNNKKIPYDWTIYYLRKKALNQRIEK

FpCas9(CN6)   GGKFIPDFYHSDLQKELEKIVRFQNQFHSEQINDKLLADIQGKSRTQTSQHFSKTLNIPL
FpCas9(CN38)  GGKFIPDFYHSDLQKELEKIVRFQNQFHSEQINDKLLADIQGKSRTQTSQHFSKTFNIPL
FpCas9(CN46)  EELAWILLNFNQKRGYYQLRGEEQEDNKNKLEEFHTLTVVDVIERE..KGKSGIWYNVIL
FcCas9        EELAWLLLQFNQKRGYNQLRDEEEESVPNKQVEFYSLKIIDVVADQPKEGRDKRWYSINL

FpCas9(CN6)   AENKGTREETKLQHYQWRNEALANELDMKILAFIITEINNQINQSSGYLGAISD......
FpCas9(CN38)  AENKGTREETKLQHYQWRNEALANELDLKILAFIITEINNQINQSSGYLGDISD......
FpCas9(CN46)  ENGWIYKRESKIALFDWIGKQKNFIVTTELNDDGTVKLNKDNEEKRSFRSPKEDDWGLVK
FcCas9        ENGWTYRRESKTPLFNWEGKTKDFIVTTDLNQDGSIKKDKEGKEKRSFRAPSEGDWTLLK

FpCas9(CN6)   ..RSKELYFNQETVGQYQYKQLQENVHAKLKNQVFYRQD...YLDEFEKIWETQAKFHSE
FpCas9(CN38)  ..RSKELYFNQETVGQYQYKQLQENVHAKLKNQVFYRQD...YLDEFEKIWETQAKFHSE
FpCas9(CN46)  IRTEQTINQSEKSVGTFIYDALLKNPNQKIKGELLRTIERKFYKDELSKILKAQIAFHHE
FcCas9        KKTEKEIYESGKTLGTYIYDALLNTPDLKIKGDFIRTIDRSIYKEELQQIINKQKEYHLE

FpCas9(CN6)   L.................NEKLKAEIR.........DITIFYQRKLKSQKHLVSFCEFEK
FpCas9(CN38)  L.................NEKLKAEIR.........DITIFYQRKLKSQKHLVSFCEFEK
FpCas9(CN46)  IFGDRNLYKKCIDELYRHNESHKNNIKDKGFDYLFLDDIIFYQRPLKSKTSLISDCSLEF
FcCas9        LNSSK.LYKACLEELYPKNEIYKSALKNKDIAYLFINDILYYQRPLKAPKYNIGKCTYES

FpCas9(CN6)   G.....................HKAIPKSSPLFQEFRIWQNLNNVVVKNETTKEVFELSE
FpCas9(CN38)  G.....................HKAIPKSSPLFQEFRIWQNLNNVVVKNETTKEVFELSE
FpCas9(CN46)  VPIKDENGILVKDDNGKQVVKPLKCIAKSNPIFQEFRLLQFVKNLKIYKKEIVNDVDVTH
FcCas9        RTFKKED.........KKIREYIPAVAKSHPLFQEFRLWQWIKNLKIYEKLSSD..NCTD

FpCas9(CN6)   ELKQLIAEELQFKESMTDVQMLTFCELKKGTHTVNFKKIEGNRTNTSIYKAFEKILEVEG
FpCas9(CN38)  ELKQLIAEELQFKESMTDVQMLTFCELKKGTHTVNFKKIEGNRTNTCIYKAFEKILEVEG
FpCas9(CN46)  DFIKTEEDLVELFNFLNDRKEIDQISLIKHLLRP..LKLKGKLLDAEVAKLRWNFVED..
FcCas9        FFLTNEQDFENLFEFLWNKKEIDHKGILEYLVKTKFPDLKPKQVKTKVKEYRWNYVYDH.

FpCas9(CN6)   YEIDFSKMKPLEIKETVAPIFETLNINTVILDFDPTIQGNDFDKQPYYQLWHLLYSAEDD
FpCas9(CN38)  YEMDFSKMKPLEIKETVAPIFETLNINTVILDFDPSIQGNDFDKQPYYQLWHLLYSAEDD
FpCas9(CN46)  ...............KIYPCNETRNQFLNAVAKID.VDKSFFDAKNTQDLWHILYSVTDK
FcCas9        ..........EKDESKTYPCGETYFLLKNKLDKIYNAQDDFLDKERLYNLWHIIYSVTDK
```

```
                            550           560           570           580           590
FpCas9(CN6)    .....DKLKETLKTKFGFKENHLPFILNINLQADYGSLSAKAIKKILPHLMDGHIYDK..
FpCas9(CN38)   .....DKLKETLKTKFGFKENHLPFLLNINLQADYGSLSAKAIKKILPHLMDGHIYDK..
FpCas9(CN46)   IEITKAITTFAKKHNLP.DEFITNFSKLKPYKKDYGSFSEKAIKKLLPLMRFGSYWKESN
FcCas9         KQYEKALAKFALKNHLDTKSFVNTFKRIPPFKNDYAIYSLKAIKKLLPLMRVGKYWNYDD

                             600
FpCas9(CN6)    .........ACLMVG......................................YNHSSSL
FpCas9(CN38)   .........ACLMAG......................................YNHSSSL
FpCas9(CN46)   IDIKTLARIDKIQTGEFDENMRTRVREKAIHLSKTTDFQGLPLWLASYIIYDRHSEASDV
FcCas9         IDPITKQKIQCIITGEENDTISTLVREKTNHFSNENDFKNLPLWLAGYIVYNSHNESSEI

               610       620           630           640           650           660
FpCas9(CN6)    TTAQNNDRVLKDTLELLKKNSLRNPVVEKILNQMINVINAIMKDPAMGKP...DEIRVEL
FpCas9(CN38)   TTAQNNDRVLKDTLELLKKNSLRNPVVEKILNQMINVINAIMKDPAMGKP...DEIRVEL
FpCas9(CN46)   SKWKTPADIQNFLKYDFKQHSLRNPIVEQVITETLRVVKDIWQHYGDGKENFFTEIHIEL
FcCas9         EKWKSPQDIDTFLK.KFKQHALRNPIVEQVVTETLRVVRDIWSKKGQGKKDFFNEIHIEL

                   670           680           690           700
FpCas9(CN6)    ARELKANNEQRKKATSDINTATTEHERIRKLLHSEFN.......................
FpCas9(CN38)   ARELKANNEQRKKATSDINAATTEHERIRKLLHSEFN.......................
FpCas9(CN46)   GREMKNDKATRERMTKKISENENTNLRIKAILTELKNDGLNDIRPYSPSQQDILKIYEEG
FcCas9         GKDLKNPVDKRRKLSSTNTKNESTNTRIKALLIELFTQGVENVKPNSPKHQEILKIFEEG

                                             710           720           730           740
FpCas9(CN6)    ...................IPRVTRNDIIRYKLWLETGGISLYTGNPIKASDLFSKNYDI
FpCas9(CN38)   ...................IPRVTRNDIIRYKLWLETGGISLYTRNAIKASDLFSKNYDI
FpCas9(CN46)   VYSNENKKEKLEEIDKIRKNAKPTPSEIIKYKLWLEQGYISPYTGKLIPLSELFTTKYQI
FcCas9         ALNSG..IEIEEDIIKISNTDQPTKKQLELYILWLEHKYKSPYTGQSIPLSKLFTPNYEI

                     750           760           770           780           790           800
FpCas9(CN6)    EHIIPKSRLFDDSFSNKTLCERQLNIDKSNKTAYSFLQEKLSENDFAAYENRVKDLFKTG
FpCas9(CN38)   EHIIPKSRLFDDSFSNKTLCERQLNIDKSNKTAYSFLQEKFSENDFAAYENRVKDLFKTG
FpCas9(CN46)   EHIIPQSRLFDDSLSNKVICESEVNELKSNMTAYEFIQNNEDRIVILTGGKTVKIFTKDA
FcCas9         EHIIPRSKYYDDNLSNKVICETEINRLKNNQLGYEFINNHEIREVTLEEGKTIRLLSPEQ

                     810                     820           830           840
FpCas9(CN6)    KIKSYTKYKKLLMADN......EIPEGFIDRQLRETQYIAKKAKEILLEVSR........
FpCas9(CN38)   KIKSYTKYKKLLMADN......EIPEGFIDRQLRETQYIAKKAKEILLEVSR........
FpCas9(CN46)   YNQHITAYYSKNKSKQKRLLSEEIPEKFIERQLNDTKYISKIVKNLLSRIVREEEEQE..
FcCas9         YWNFVTTYYAKNKAKRENLLLKELPVQMVEKQLNDTRYVSKYVMQLLSKIVRKEDGTDNG

                   850           860           870           880           890           900
FpCas9(CN6)    ....NVTATIGSVTDKLRQDWELVDVMKELNWEKYDKLGLTHIEEGKNGERLSKIKD...
FpCas9(CN38)   ....NVTATIGSVTDKLREDWELVDVMKELNWEKYDKLGLTHIEEGKNGERLSKIKD...
FpCas9(CN46)   VTSKHIVSLNGSITSRMKQDWGLNDVWNNIVTPRFERLNALTNSNDFGQFELKKDAYGNK
FcCas9         INSTNILSISGKMTRALKNDWDLNDVWNDLLLYRFERLNTITKTNLFTTYSEQKQKR...

                                       910           920           930           940
FpCas9(CN6)    .................WTKRNDHRHHAMDAITVAFTKPAYIQYLNNLNAKTQGENK...
FpCas9(CN38)   .................WTKRNDHRHHAMDAITVAFTKPAYIQYLNNLNAKTQGENK...
FpCas9(CN46)   GKQIFQTTVPDAIAKGFTKKRIDHRHHALDALVIACVSRTHINYLNNLNARDTDDKA...
FcCas9         .....MPIVPDQYAKGFHKKRLDHRHHALDAIVIACITRNHINYMNNQNIQGEKLKKNAI

                                     950           960           970           980
FpCas9(CN6)    ...............ANSIIGIENKHLYRDKNNKLRFISPMKNFREEAKKQLESILISYK
FpCas9(CN38)   ...............ANSIIGIENKHLYRDKNNKLRFISPMKNFREEAKKQLESILISYK
FpCas9(CN46)   ...........VKHELRNKLCFKTKPDSN.GNYKWEFYKPWKDFTTEAEDKLNTTIVSFK
FcCas9         EKENYREILQKSRLDIRAQLCEKKYNDNDPTKYNWVFIKPWADFTENTKDILESIIPSFK
```

```
                  990       1000      1010      1020      1030      1040
FpCas9(CN6)    AKNKVVTKNKNTTKKSGGTNQKIQLTPRGRLHKETVYGKLQQYATKEEKVNASFTEDYIQ
FpCas9(CN38)   AKNKVVTKNKNTTKKSGGTNQKIQLTPRGRLHKETVYGKLQQYATKEEKVNASFTEDYIQ
FpCas9(CN46)   QNTRVINKTVNKYTNYKDEKGNLNIGTNGLVEKKIITQTKGDNWAIRKPMHAETVSGKVF
FcCas9         QNLRIINKTVNYYEKYN.ENG..........KKVKVRQIKGDNWAIRKPLHKETISGKVH

               1050      1060      1070                     1080      1090
FpCas9(CN6)    KVAKKEYREALLKRLEENDNDPKKAFTGKNALN..............KTPIYISLKDNII
FpCas9(CN38)   KVAKKEYREALLKRLEENDNDPKKAFTGKNALN..............KTPIYISLKDNII
FpCas9(CN46)   LKRIKQSPITIANAIEQIEFIVDKEVKKQLASKIKQYPNNMVGLKKHLKAFPVMIDGKAI
FcCas9         LSYKTPAKGKIL................................................

                   1100      1110       1120      1130      1140
FpCas9(CN6)    VPEKVKTVWLETDYTIRKDITPDLK.IDKVIDVGIKRILQNRLNEFNGD.......PKKA
FpCas9(CN38)   VPEKVKTVWLETDYTIRKDITPDLK.IDKVIDVGIKRILQNRLNEFNGD.......PKKA
FpCas9(CN46)   DKVQVYETIEATATRKTLDITFDEKKIEKITDTGIQKILLNHLKQEMYQNAIHENGEKIP
FcCas9         ...........TATRKVLDSSMDFKSIEKITDTGIQKILKNYLEYKKSP...........

                 1150      1160      1170      1180      1190      1200
FpCas9(CN6)    FVNLGESPIWLNKEKGIAIKRVTISGVSNAQALHTKKDHLGNEILDKNGNPIPVDFISTG
FpCas9(CN38)   FVNLGESPIWLNKEKGIAIKRVTISGVSNAQALHTKKDHLGNEILDKNGNPIPVDFVSTG
FpCas9(CN46)   AHEVAFSENGLDELNKNLTTLNNGKKHQPIKKVRVFEEGGKFRLGQTQNKADKYVEAAKG
FcCas9         ..ELAFSPEGIEELNATITKYNNGFHHKPIYKVRIYEEGVKFPLGNKGNKKSKYVEAEKG

                 1210      1220      1230      1240      1250      1260
FpCas9(CN6)    NNHHVAIYRDEKGNLQEEVVSFYDAVIRRNLGLSVINKNHEKGWEFLFSMKQNEFFIFPS
FpCas9(CN38)   NNHHVAIYRDEKGNLQEEVVSFYDAVIRRNLGLSVINKNHEKGWEFLFSMKQNEFFIFPS
FpCas9(CN46)   TNLFFAIYQDEKGKRNYKTIPFNEVIERQKQGLSAAQETDENGNRLLFTLSPNDLVYLPT
FcCas9         TNLFFAIYKDQENNRYFETIPFNDVLETLKKGMSAVPEKNQEENTLVFALSPQDLVFVPT

                 1270      1280      1290                1300      1310
FpCas9(CN6)    DGFNPHEIDLLNPNNYHLISPNIFRVQKISTKNY........MFNHHLETKAVDGEMLKS
FpCas9(CN38)   DDFNPHEIDLLNPNNYHLISPNMFRVQSLSVVQYGNSTIRDFKFRHHLETTVED......
FpCas9(CN46)   EDEKENLTTINFDKLSKEQTERIYKFVSCTSGEGHFVSNNYSKEIISNENGSNNKNERML
FcCas9         LDEIENPDIVDFKNLTKDQTNRIYKMVSSSGKQCFFIKAEVATSIVN...........KM

               1320      1330       1340      1350
FpCas9(CN6)    KKELSKTSYHFIQTPTNLN.GIIKVRINHLGKIVHLGEY
FpCas9(CN38)   KKELTNITYKQIKSLIPLN.EIIKVRINHLGKIVHVGEY
FpCas9(CN46)   ELNNSNTIYDEKEKPVMIKSVCWKLEVDRLGKIRKIIR.
FcCas9         EFSTSNKMENSIEG.IQIKSNCWKLKLDRLGTITRVIK.
```
