# Supplementary material for: Identification of a novel type II-C Cas9 from the fish pathogen Flavobacterium psychrophilum
Source: Front Microbiol. 2023 Jun 15;14:1181303. doi: 10.3389/fmicb.2023.1181303 (PMC10309648; doi:10.3389/fmicb.2023.1181303)
Supplement: Supplementary file 4 [file Data_Sheet_2.PDF]

Fig. S2 The C-terminus alignment of FpCas9s from *F. psychrophilum* strains CN06, CN38, 97708 and FI070.

```
CN6    DWTKRNDHRHHAMDAITVAFTKPAYIQYLNNLNAKTQ........GENKANSIIGIENKH
CN38   DWTKRNDHRHHAMDAITVAFTKPAYIQYLNNLNAKTQ........GENKANSIIGIENKH
97708  DWTKRNDHRHHAMDAITVAFTKPAYIQYLNNLNAKTQ........GENKAYSIIGIEEKY
FI070  DWTKRNDHRHHAMDAITVAFTKPAYIQYLNNLNARSINIKEISEATDKKSKIIIGIETKY

CN6    LYRDKNNKLRFISPMKNFREEAKKQLESILISYKAKNKVVTKNKNTTKKSGGTNQKIQLT
CN38   LYRDKNNKLRFISPMKNFREEAKKQLESILISYKAKNKVVTKNKNTTKKSGGTNQKIQLT
97708  LKRDKNNKLRFIPPMKSFREEAKKQLESILISYKAKNKVVAKNKNTTKKAGGTNQKIQLT
FI070  LERDKNNKLRFIPPMENFREEAKKQLESILISYKAKNKVVTKNKNITKKSGGTNQKIQLT

CN6    PRGRLHKETVYGKLQQYATKEEKVNASFTEDYIQKVAKKEYREALLKRLEENDNDPKKAF
CN38   PRGRLHKETVYGKLQQYATKEEKVNASFTEDYIQKVAKKEYREALLKRLEENDNDPKKAF
97708  PRGQLHKETVYGKLQRYETKEEKVNASFTEDYIQKVAKKEYREALLKRLEENGNDPKKAF
FI070  PRGQLHKETVYGKLQQYVTKEEKVNANFTAEYIQKVAKKEYSEALLKRLLENDNDPKKAF

CN6    TGKNALNKTPIYISLKDNIIVPEKVKTVWLETDYTIRKDITPD....LK.IDKVIDVGIK
CN38   TGKNALNKTPIYISLKDNIIVPEKVKTVWLETDYTIRKDITPD....LK.IDKVIDVGIK
97708  TGKNALNKTPIYISLKDNIVVPEKVKTVWFEDNYTIRKDITPDNFKDLKSIAKVMDSGLK
FI070  TGKNTLNKTPIYISLKDNIVVPEKVKIVWLEADYTIRKDITPD....LK.IDKVIDIGLK

CN6    RILQNRLNEFNGDPKKAFVNLGESPIWLNKEKGIAIKRVTISGVSNAQALHTKKDHLGNE
CN38   RILQNRLNEFNGDPKKAFVNLGESPIWLNKEKGIAIKRVTISGVSNAQALHTKKDHLGNE
97708  TILEDRLAEYGNDPKKAFMNLEENPIWQNKEKGIAIKRVTISGVSKTESLHNKKDHLGNE
FI070  AILQDRLAEFGNDPKKAFINLDENPIWQNKEKGIAIKRVTISGVSNAQALHTKKDHFGNE

CN6    ILDKNGNPIPVDFISTGNNHHVAIYRDEKGNLQEEVVSFYDAVIRRNLGLSVINKNHEKG
CN38   ILDKNGNPIPVDFVSTGNNHHVAIYRDEKGNLQEEVVSFYDAVIRRNLGLSVINKNHEKG
97708  ILDKDGKTIPVDFVSTGNNHHVAIYKDEKGNLQEEVVSFYDAVVLAKEGLPVVNKNHKNG
FI070  ILDENGNPIPVDFVSTGNNHHVAIYKDEKGNLQEEVVSFYDAVVRRNLGLSVINKNHEKG

CN6    WEFLFSMKQNEFFIFPSDGFNPHEIDLLNPNNYHLISPNIFRVQKISTKNY........M
CN38   WEFLFSMKQNEFFIFPSDDFNPHEIDLLNPNNYHLISPNMFRVQSLSVVQYGNSTIRDFK
97708  WEFLFSMKQNEFFIFPSDGFNPHEIDLLNPNNYHLISPNIFRVQKISTKDY........F
FI070  WKFLFSMKQNEFFIFPSDGFNPHEIDLLNPNNYHLISPNMFRVQKISTKDY........F

CN6    FNHHLETKAVDGEMLKSKKELSKTSYHFIQTPTNLNGIIKVRINHLGKIVHLGEY
CN38   FRHHLETTVED......KKELTNITYKQIKSLIPLNEIIKVRINHLGKIVHVGEY
97708  FRHHLETTVED......KKELKDISFKRLG.INGINNITKVRINHLGQIVHLGEY
FI070  FRHHLETTVED......KKELKEISFKRLG.INGINNITKVRINHLGKIVHLGEY
```
