# Supplementary material for: Identification of a novel type II-C Cas9 from the fish pathogen Flavobacterium psychrophilum
Source: Front Microbiol. 2023 Jun 15;14:1181303. doi: 10.3389/fmicb.2023.1181303 (PMC10309648; doi:10.3389/fmicb.2023.1181303)
Supplement: Supplementary file 5 [file Data_Sheet_3.PDF]

Fig. S3 The C-terminus alignment of FpCas9s from *F. psychrophilum* strains CN46, 8888, FPS-R9 and NO098.

```
        1        10        20        30        40        50        60
NO098   QTTVPDAIAKGFTKKRIDHRHHALDALVIACVSRTHINYLNNLNARDTDDKAVKHELRNK
FPS-R9  QTTVPDAIAKGFTKKRIDHRHHALDALVIACVSRTHINYLNNLNARDTDDKAVKHELRNK
CN46    QTTVPDAIAKGFTKKRIDHRHHALDALVIACVSRTHINYLNNLNARDTDDKAVKHELRNK
8888    QTTVPDAIAKGFTKKRIDHRHHALDALVIACVSRTHINYLNNLNARDTDDKAVKHELRNK

                 70        80        90       100       110       120
NO098   LCFKTKPDSNGNYKWEFYKPWKDFTTEAEDKLNTTIVSFKQNTRVINKTVNKYTNYKDEK
FPS-R9  LCFKTKPDSNGNYKWEFYKPWKDFTTEAEDKLNTTIVSFKQNTRVINKTVNKYTNYKDEK
CN46    LCFKTKPDSNGNYKWEFYKPWKDFTTEAEDKLNTTIVSFKQNTRVINKTVNKYTNYKDEK
8888    LCFKTKPDSNGNYKWEFYKPWKDFTTEAEDKLNTTIVSFKQNTRIINKTVNKYTNYKDEK

                130       140       150       160       170       180
NO098   GNLNIGTNGLVEKKIITQTKGDNWAIRKPMHAETVSGKVFLKRIKQSPITIANAIEQIEF
FPS-R9  GNLNIGTNGLVEKKIITQTKGDNWAIRKPMHAETVSGKVFLKRIKQSPITIANAIEQIEF
CN46    GNLNIGTNGLVEKKIITQTKGDNWAIRKPMHAETVSGKVFLKRIKQSPITIANAIEQIEF
8888    GNLNIGTNGLVEKKIITQTKGDNWAIRKPMHAETVSGKVFLKRIKQSPITIANAIEQIEF

                190       200       210       220       230       240
NO098   IVDKEVKKQLASKIKQYPNNMVGLKKHLKAFPVMIDGKAIDKVQVYETIEATATRKTLDN
FPS-R9  IVDKEVKKQLASKIKQYPNNMVGLKKHLKAFPVMIDGKAIDKVQVYETIEATATRKTLDN
CN46    IVDKEVKKQLASKIKQYPNNMVGLKKHLKAFPVMIDGKAIDKVQVYETIEATATRKTLDI
8888    IVDKEVKKQLASKIKQYPNNMVGLKKHLKAFPVMIDGKAIDKVQVYETIEATATRKTLDI

                250       260       270       280       290       300
NO098   SFDEKTINSITDTGIQKILLNHLKQEMYQNAIDENGKKIPPHESAFSEYGLEMLNKNIKK
FPS-R9  SFDEKTINSITDTGIQKILLNHLKQEMYQNAIDENGKKIPPHESAFSEYGLEMLNKNIKK
CN46    TFDEKKIEKITDTGIQKILLNHLKQEMYQNAIHENGEKIPAHEVAFSENGLDELNKNLTT
8888    TFDEKKIEKITDTGIQKILLNHLKQEMYQNAIDENGEKIPAHEVAFSENGLDELNKNLTT

                310       320       330       340       350       360
NO098   LNNGKAHQPIKKVRVFMEGTGKFAVGTNGNKKDKYVVAAKGTNLFFAIYQDEKGKRNYKT
FPS-R9  LNNGKAHQPIKKVRVFMEGTGKFAVGTNGNKKDKYVVAAKGTNLFFAIYQDEKGKRNYKT
CN46    LNNGKKHQPIKKVRVFEEG.GKFRLGQTQNKADKYVEAAKGTNLFFAIYQDEKGKRNYKT
8888    LNNGKKHQPIKKVRVFEES.NKFPLGETGNKKNKFVETAKGTNLFFAIYQDDLNNKKFIT

                370       380            390       400       410
NO098   IPFNEVIERQKQSLSAAQETDENGN.....RLLFTLSPNDLVYLPTEDEKENLTTINFDK
FPS-R9  IPFNEVIERQKQSLSAAQETDENGN.....RLLFTLSPNDLVYLPTEDEKENLTTINFDK
CN46    IPFNEVIERQKQGLSAAQETDENGN.....RLLFTLSPNDLVYLPTEDEKENLTTINFDK
8888    IPLNIVIQNLKDGLSPVPDKYLDNKTKVEYKLTQFLSPNDLVYTPTNDELENSNQININN

           420       430       440       450                 460
NO098   LSKEQVLRVLKMVSFTGNQAFFVSNNIATSIVDKMEFS........ALNKMEKSIDG...
FPS-R9  LSKEQVLRVLKMVSFTGNQAFFVSNNIATSIVDKMEFS........ALNKMEKSIDG...
CN46    LSKEQTERIYKFVSCTSGEGHFVSNNYSKEIISNENGSNNKNERMLELNNSNTIYDEKEK
8888    FNKDQVCRLYNVNDFSGYTIYFTPNQFAKNIAPKELDTS.......FDSKLSKSLEG...

              470       480
NO098   .TMIKSLCLKLEVDRLGKIKKIIR
FPS-R9  .TMIKSLCLKLEVDRLGKIKKIIR
CN46    PVMIKSVCWKLEVDRLGKIRKIIR
8888    .ITIKENFWKLEVDRLGKIKKIIR
```
